# Combined QTL mapping on bi-parental immortalized heterozygous populations to detect the genetic architecture on heterosis

**DOI:** 10.3389/fpls.2023.1157778

**Published:** 2023-04-04

**Authors:** Xuexue Huo, Jiankang Wang, Luyan Zhang

**Affiliations:** ^1^ National Key Facility for Crop Gene Resources and Genetic Improvement, and Institute of Crop Sciences, Chinese Academy of Agricultural Sciences (CAAS), Beijing, China; ^2^ National Nanfan Research Institute (Sanya), Chinese Academy of Agricultural Sciences (CAAS), Sanya, Hainan, China

**Keywords:** immortalized population, pure-line population, QTL mapping, combined analysis, heterosis

## Abstract

From bi-parental pure-inbred lines (PIL), immortalized backcross (i.e., IB_1_ and IB_2_, representing the two directions of backcrossing) and F_2_ (i.e., IF_2_) populations can be developed. These populations are suitable for genetic studies on heterosis, due to the present of both homozygous and heterozygous genotypes, and in the meantime allow repeated phenotyping trials across multiple locations and years. In this study, we developed a combined approach of quantitative trait locus (QTL) mapping, when some or all of the four immortalized populations (i.e., PIL, IB_1_, IB_2_, and IF_2_) are available. To estimate the additive and dominant effects simultaneously and accurately, suitable transformations are made on phenotypic values from different populations. When IB_1_ and IB_2_ are present, summation and subtraction are used. When IF_2_ and PIL are available, mid-parental values and mid-parental heterosis are used. One-dimensional genomic scanning is performed to detect the additive and dominant QTLs, based on the algorithm of inclusive composite interval mapping (ICIM). The proposed approach was applied to one IF_2_ population together with PIL in maize, and identified ten QTLs on ear length, showing varied degrees of dominance. Simulation studies indicated the proposed approach is similar to or better than individual population mapping by QTL detection power, false discovery rate (FDR), and estimated QTL position and effects.

## Introduction

Heterosis, also known as hybrid vigor, is a phenomenon that the performance of hybrids outperforms their parents for one or more traits. Over the past 100 years, hybrid breeding has been proved to be highly successful in exploiting the heterosis in a number of crop species, and has made great contributions to agricultural production (Whitford et al., 2013; Labroo et al., 2021). The rapid development of molecular technology is expected to deepen our understanding on heterosis. Conventional bi-parental populations, such as backcross (BC), F_2_ and F_2:3_, can be used to study the dominance-related genetic effects included in heterosis. However, these populations cannot be phenotyped in multi-environmental trials, and thus the analysis for QTL stability and QTL by environment interaction are not possible (Wang et al., 2020). To avoid the problems in conventional heterozygous populations, the concept of immortalized heterozygous populations has been proposed.

Immortalized heterozygous populations are derived from a population of bi-parental pure-inbred lines (PIL population). Immortalized BC (IBC) is generated by the hybridization between PIL with the two original inbred parents, similar to backcrossing the F_1_ hybrid. The backcrossing of the pure lines with the first parent is denoted by IB_1_, and backcrossing of the pure lines with the second parent is denoted by IB_2_. Pure lines in PIL can be derived either by doubled haploids (DH) technology or repeated selfing, since the F_1_ hybrid derived from two homozygous parents. Pure inbred lines generated by repeated selfing are called recombination inbred lines (RIL). Immortalized F_2_ (IF_2_) is generated by the hybridization between two lines in PIL, similar to selfing the F_1_ hybrid. As each line in PIL can be maintained by selfing, IB_1_, IB_2_ and IF_2_ can be repeatedly produced whenever needed just like any typical F_1_ hybrids, which is the reason to be called ‘immortalized’. Due to their repeatability, populations IBC and IF_2_ can be evaluated in multi-environmental trials with replications. In the sense of selfing maintenance, PIL can be called ‘immortalized’ as well. If IB_1_, IB_2_ and IF_2_ are called the immortalized heterozygous populations, PIL may be called the immortalized homozygous population. Therefore, in this study, PIL is occasionally called the immortalized homozygous population, and IB_1_, IB_2_ and IF_2_ are occasionally called the immortalized heterozygous populations, so as to reflect the genetic constitutions of these populations. Genotypes of pure lines are only needed in PIL; those of hybrids in IB_1_, IB_2_ and IF_2_ can be inferred from their respective parents in PIL and the two original parents.

Over the past decades, a number of immortalized heterozygous populations have been developed in several crop species, and used for genetic analysis on quantitative traits and the study on the genetic mechanism of heterosis, such as rice (Hua et al., 2002; Mei et al., 2005; Zhou et al., 2012), maize (Guo et al., 2014; Yi et al., 2019), wheat (Yuan et al., 2012), cotton (Liu et al., 2014; Wang et al., 2016; Li et al., 2018a; Ma et al., 2019), *Brassica juncea* (Aakanksha et al., 2021), and rapeseed (Liu et al., 2017). Conventional QTL mapping methods have been applied in these populations, including interval mapping (IM; Lander and Botstein, 1989), composite interval mapping (CIM; Zeng, 1994), and inclusive composite interval mapping (ICIM; Li et al., 2007; Zhang et al., 2008; Wang, 2009). As examples, Mei et al. (2005) developed two-directional IBC populations from bi-parental RILs and applied IM to investigate the gene action types on seven quantitative traits in rice, including heading date, plant height, and panicle length and so on. Li et al. (2018a) developed IBC and IF_2_ populations in upland cotton, and applied CIM to detect heterotic loci related to fiber quality traits. Yi et al. (2019) conducted QTL mapping for yield-related traits in maize IF_2_ and RIL populations based on ICIM algorithm. Previous studies showed that the genetic basis of heterosis is more likely to be a combination of various genetic effects, such as additive, partial dominant, over-dominant, and epistatic effects (Zhan et al., 2016; Liu et al., 2020; Ouyang et al., 2022), indicating highly complicated nature of the heterosis phenomenon in biology.

Originated from the same two original parents, immortalized heterozygous populations are highly related. In previous studies, they were treated as conventional bi-parental populations, and analyzed individually without considering their close relationship. The combined analysis with pure lines and their derived immortalized heterozygous populations takes into consideration more correlated genetic information simultaneously, and therefore improves mapping accuracy. Our objectives in this study were: (1) to present the algorithm of combined QTL mapping approach; (2) to apply the proposed approach in an actual maize population; (3) to demonstrate its efficiency by comparison with the individual population mapping through simulation studies.

## Materials and methods

### Immortalized heterozygous populations used for QTL mapping

**Figure 1:**
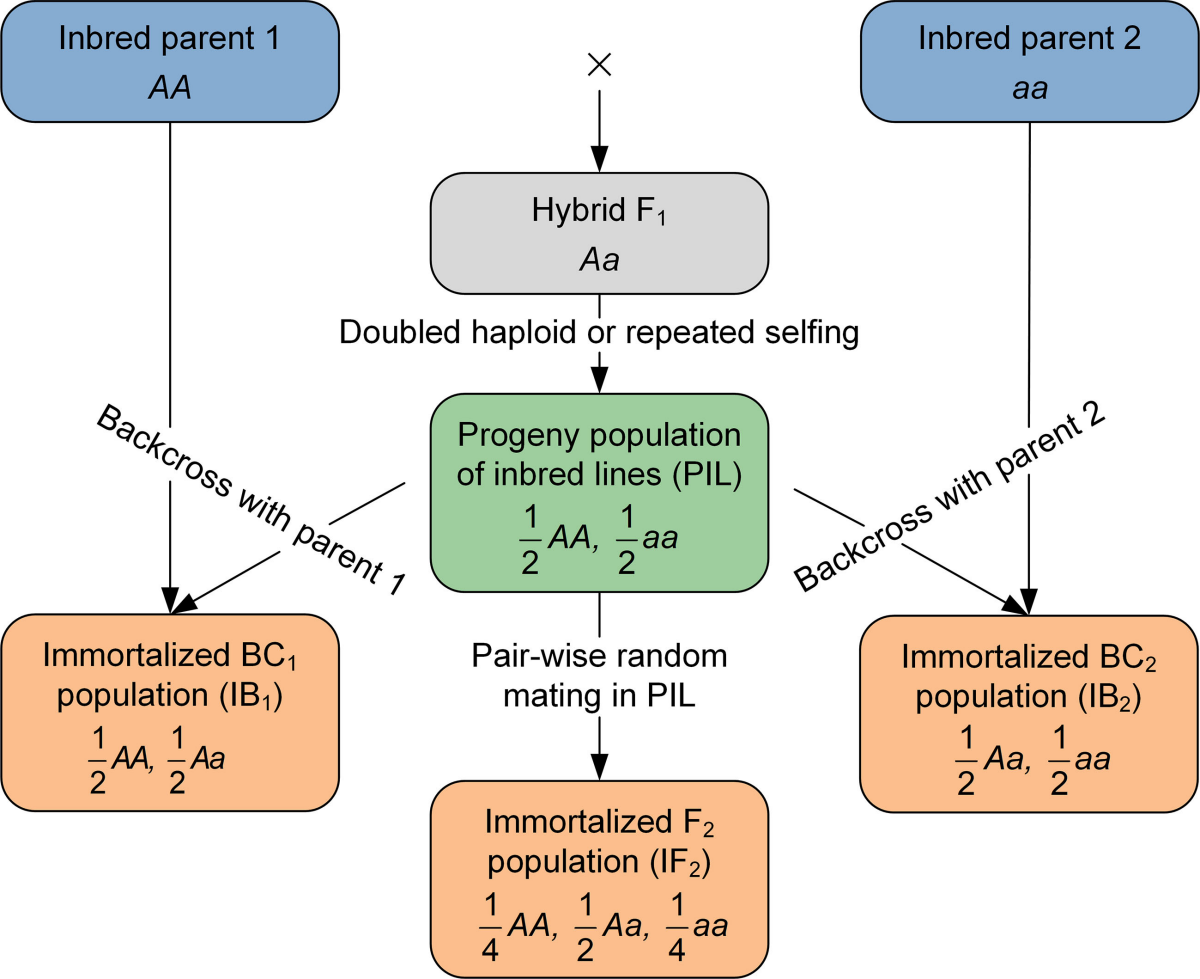
Diagram of the development procedure of immortalized backcross and immortalized F_2_ populations.

The combined mapping approach depends on multiple immortalized populations, which can be some or all of four populations, i.e., PIL, IB_1_, IB_2_, and IF_2_. Relationship between the four populations is shown in **Figure 1** (see also Zhang et al., 2022b). In one-directional IBC population, only genotypes of the recurrent parent and hybrid F_1_ are present. Additive and dominant effects cannot be estimated simultaneously, unless the two-directional IBC populations are considered together. Genotypic composition and segregation ratio in IF_2_ are similar to conventional F_2_ at individual genetic loci. As far as two linked loci are considered, **Supplementary Tables 1** and **2** give the genotypes and their frequencies in two types of PIL. If estimated in the DH-derived IBC or IF_2_, recombination frequency would be exactly the same as that estimated in conventional BC or F_2_ populations. However, if estimated in the RIL-derived IBC or IF_2_, recombination frequency would represent the accumulated crossing-over rate during the repeated selfing (Wang et al., 2017).

### One-locus genetic model and effects in the four immortalized populations

Assume the mean values of three genotypes (i.e., *AA*, *Aa* and *aa*) at one bi-allelic locus are represented by *m*+*a*, *m*+*d* and *m*-*a*, respectively, where *m*, *a*, and *d* are the mid-parental value, additive and dominant effects at the locus. When no segregation distortion is considered, two genotypes *AA* and *aa* have equal frequency at 0.5 in PIL, allowing the estimation of additive effect between two homozygous genotypes (**Table 1**). Genotypes *AA* and *Aa* have equal frequency at 0.5 in IB_1_, with a difference equal to *a*-*d* in genotypic values. Genotypes *Aa* and *aa* have equal frequency at 0.5 in IB_2_, with a difference equal to *a+d* (**Table 1**). Therefore, additive and dominant effects cannot be separated in either population, which can also be seen from the genetic variance given in **Table 1**. However, genotype *AA* in IB_1_ and genotype *Aa* in IB_2_ are both derived from genotype *AA* in PIL, and half of the summation of the two genotypic values is equal to $m+\frac{1}{2}\left(a+d\right)$ . Genotype *Aa* in IB_1_ and genotype *aa* in IB_2_ are both derived from genotype *aa* in PIL, and half of the summation of the two genotypic values is equal to$m+\frac{1}{2}\left(d-a\right)$ . Denote the summation transformation as

**Table 1:** Genotypes, genotypic values and genetic variances at one locus in populations PIL, IB_1_ and IB_2_, where *m*, *a*, and *d* are mid-parental value, additive and dominant effects, respectively.

Parameters	Immortalized populations			Transformations	
	PIL	IB_1_	IB_2_	$\frac{1}{2}\left({\text{IB}}_{1}+{\text{IB}}_{2}\right)$	$\frac{1}{2}\left({\text{IB}}_{1}-{\text{IB}}_{2}\right)$
Two genotypes and genotypic values	*AA*, m+a	*AA*, m+a	*Aa*, m+d	$m+\frac{1}{2}\left(a+d\right)$	$\frac{1}{2}\left(a-d\right)$
	*aa*, m−a	*Aa*, m+d	*aa*, m−a	$m+\frac{1}{2}\left(d-a\right)$	$\frac{1}{2}\left(a+d\right)$
Difference between the two genotypes	2a	a−d	a+d	a	−d
Genetic variance	$a^{2}$	$\frac{1}{4}{\left(a-d\right)}^{2}$	$\frac{1}{4}{\left(a+d\right)}^{2}$	$\frac{1}{4}a^{2}$	$\frac{1}{4}d^{2}$

No segregation distortion is considered.


(1) $$S=\frac{1}{2}\left({\text{IB}}_{1}+{\text{IB}}_{2}\right)$$

Difference between the two values from transformation *S* is equal to *a*, i.e., $\left(m+\frac{1}{2}\left(a+d\right)\right)-\left(m+\frac{1}{2}\left(d-a\right)\right)=a$ . Similarly, denote the subtraction transformation as


(2) $$T=\frac{1}{2}\left({\text{IB}}_{1}-{\text{IB}}_{2}\right)$$

Difference between the two values from transformation *T* can be found to be equal to -*d*, i.e., $\frac{1}{2}\left(a-d\right)-\frac{1}{2}\left(a+d\right)=-d$ . Therefore, when IB_1_ and IB_2_ are both available, the summation and subtraction transformations can separate additive and dominant effects included in the one-locus genetic model (**Table 1**).

When no segregation distortion is considered, three genotypes *AA*, *Aa* and *aa* have frequencies at 0.25, 0.5 and 0.25 in IF_2_, allowing the estimation of mid-parental value, additive and dominant effects simultaneously (**Table 2**). When PIL is available, mid-parent value can be calculated, and denoted as

**Table 2:** Genotypes, genotypic values and genetic variances at one locus in population IF_2_, where *m*, *a*, and *d* are mid-parental value, additive and dominant effects, respectively.

PIL_1_	PIL_2_	Frequency	IF_2_	Transformations	
				$\frac{1}{2}\left({\text{PIL}}_{1}+{\text{PIL}}_{2}\right)$	${\text{IF}}_{2}-\frac{1}{2}\left({\text{IB}}_{1}+{\text{IB}}_{2}\right)$
*AA*, m+a	*AA*, m+a	$\frac{1}{4}$	*AA*, m+a	m+a	0
*AA*, m+a	*aa*, m−a	$\frac{1}{2}$	*Aa*, m+d	m	d
*aa*, m−a	*aa*, m−a	$\frac{1}{4}$	*aa*, m−a	m−a	0
Genetic variance			$\frac{1}{2}a^{2}+\frac{1}{4}d^{2}$	$\frac{1}{2}a^{2}$	$\frac{1}{4}d^{2}$

No segregation distortion is considered.


(3) $$M=\frac{1}{2}\left({\text{PIL}}_{1}+{\text{PIL}}_{2}\right)$$

Genotype *AA* in IF_2_ is generated by the cross between genotype *AA* in PIL_1_ and genotype *AA* in PIL_2_; *Aa* in IF_2_ is generated by the cross between *AA* (or *aa*) in PIL_1_ and *aa* (or *AA*) in PIL_2_; and *aa* in IF_2_ is generated by the cross between *aa* in PIL_1_ and *aa* in PIL_2_. Therefore, the mid-parental values are equal to *m*+*a*, *m*, and *m*-*a* for the three genotypes *AA*, *Aa* and *aa* in IF_2_, respectively. When PIL and IF_2_ are both available, mid-parental heterosis of any F_1_ hybrid in IF_2_ can be defined as well, i.e., the difference of F_1_ hybrid from the mean of its parents in PIL, and denoted as


(4) $$H={\text{IF}}_{2}-\frac{1}{2}\left({\text{PIL}}_{1}+{\text{PIL}}_{2}\right)$$

Mid-parental heterosis can be found to be equal to 0, *d* and 0 for the three genotypes *AA*, *Aa* and *aa* in IF_2_, respectively. Therefore, additive and dominant effects can be separated by the mid-parental value and mid-parental heterosis, which can also be seen from the genetic variance given in **Table 2**.

### Combined QTL mapping approach with immortalized populations

The combined mapping using populations IB_1_ and IB_2_ is named IBC; using populations IF_2_ and PIL is named IFL; using populations IB_1_, IB_2_, and PIL is named IBL; using populations IB_1_, IB_2_, and IF_2_ is named IBF; using populations IB_1_, IB_2_, IF_2_, and PIL is named BFL (**Table 3**). When populations IBC and PIL are available, IBC and IBL can be conducted; when populations IF_2_ and PIL are available, IFL can be conducted; when all the four populations are available, IBF and BFL can be conducted. Populations IB_1_, IB_2_, and IF_2_ can also be analyzed independently, and these individual population mappings are named IB1, IB2 and IF2, respectively (**Table 3**). Both independent and combined mapping approaches are based on the ICIM algorithm. To separate additive and dominant effects, summation and subtraction transformations (Eqs. 1 and 2) are used in combined mappings IBC, IBL, IBF and BFL. Mid-parental value and mid-parental heterosis are used in combined mappings IFL and BFL (Eqs. 3 and 4; **Table 3**).

**Table 3:** Naming and properties of individual and combined QTL mappings, depending on available populations.

Category of mapping	Naming	Population(s) needed	Transformations	Effects to be estimated
Individual mapping	IB1	IB_1_	None	a−d
	IB2	IB_2_	None	a+d
	IF2	IF_2_	None	a and d
Combined mapping	IBC	IB_1_ and IB_2_	Summation and subtraction	a and d
	IFL	IF_2_ and PIL	Mid-parental value and mid-parental heterosis	a and d
	IBL	IB_1_, IB_2_ and PIL	Summation and subtraction	a and d
	IBF	IB_1_, IB_2_ and IF_2_	Summation and subtraction	a and d
	BFL	IB_1_, IB_2_, IF_2_ and PIL	Summation, subtraction, mid-parental value and mid-parental heterosis	a and d

### Algorithm of the combined QTL mapping approach

Compared with the other existing mapping methods, ICIM simplifies the genetic background control and improves the efficiency of QTL detection, which has been widely used in bi-parental populations (Li et al., 2007; Zhang et al., 2008; Wang, 2009; Meng et al., 2015), hybrid F_1_ from two heterozygous parents (Zhang et al., 2015a; Zhang et al., 2015b), and multi-parental populations (Zhang et al., 2017; Shi et al., 2019; Zhang et al., 2019). Mapping algorithm on individual populations has been covered in previous publications. As an example, IBC is used here to illustrate the combined mapping approach. First, a linear regression model is built in each population, similar to the algorithm implemented in software package QTL IciMapping (Li et al., 2007; Zhang et al., 2008; Meng et al., 2015), i.e.,


, (5) $$y_{ih}=b_{0h}+\sum_{j=1}^{m+1}b_{jh}x_{ij}+\epsilon_{ih}$$

where *y_ih_* is the phenotypic value of the *i* ^th^ individual in the *h* ^th^ population (*h*=1, 2 in IBC); *b*_0_ *_h_* is the overall mean of the linear model, and *b_jh_* is the partial regression coefficient of phenotype on the *j* ^th^ marker in the *h* ^th^ population (*h*=1, 2); *x_ij_* is the indicator of the *j* ^th^ marker genotype for the *i* ^th^ individual in PIL, valued at 1 and -1 for the two parental types; $\epsilon_{ih}$ is the residual random error, following a normal distribution with a mean of zero. Then, stepwise regression is performed on the phenotypes of each population to identify significant markers in Eq. (5).

For a testing position in marker interval [*k*, *k*+1], phenotypic value of the *i* ^th^ individual in the *h* ^th^ population is adjusted by Eq. 6, i.e.,


(6) $$\Delta y_{ih}=y_{ih}-\sum_{j\neq k,k+1}{\hat{b}}_{jh}x_{ij},h=1,2$$

where ${\hat{b}}_{jh}$ is the estimate of *b_jh_* for significant markers identified by stepwise regression in linear model Eq. 5. Summation and subtraction transformations (Eqs. 1 and 2) are conducted on adjusted phenotypic values, i.e., $\begin{array}{ll} S_{i}=\frac{1}{2}\left(\Delta y_{i1}+\Delta y_{i2}\right), & T_{i}=\frac{1}{2} \end{array}\left(\Delta y_{i1}-\Delta y_{i2}\right)$ . QTL position and effect information in the current interval is contained in the transformed phenotypic values *S_i_* and *T_i_*, which are not changed until the testing position moves to the next marker interval. Finally, conventional interval mapping is conducted on *S_i_* and *T_i_* to detect additive and dominant QTLs, respectively.

The following null and alternative hypotheses are used to test the existence of QTL at the current scanning position, i.e.,

*H*_0_: $\mu_{1h}=\mu_{2h}$ (*h*=1, 2);

*H*_1_: non-*H*_0_, i.e., in at least one transformation, $\mu_{1h}\neq \mu_{2h}$ ; where $\mu_{1h}$ and $\mu_{2h}$ are the average genotypic values of two genotypes at the tested position in the *h* ^th^ transformation. The likelihood ratio of hypotheses *H*_1_ versus *H*_0_ is denoted by LOD*_S_* and LOD*_T_* for phenotypic values *S_i_* and *T_i_*, respectively. The existence of QTL can be tested by a weighted average of the two LOD scores, where the weights are determined by the least square method. Relationship between LOD scores is given in **Supplementary Table 3** for each combined mapping. Detection of QTL depends on total LOD score which is equal to the sum of LOD scores indicating the significance of additive and dominant effects, i.e., LOD_A_ and LOD_D_. LOD scores from individual populations IB_1_, IB_2_, and IF_2_ are calculated directly, the same as those in Li et al. (2007) and Zhang et al. (2008).

### Actual PIL and immortalized F_2_ populations in maize

The PIL population in maize consists of 166 RILs, which were derived from an elite hybrid variety Yuyu22 showing significant heterosis. Two inbred parents of Yuyu22 were Zong3 and 87-1, coming from two heterotic groups. The maize IF_2_ population with a size of 157 was constructed by hybridization between the 166 RILs (Guo et al., 2014). The RILs were sequenced by a maize SNP50 genotyping chip. A total of 3184 bins were treated as markers to construct the genetic linkage map after merging 18840 SNPs (Guo et al., 2014). Ear length (EL) in the two populations was measured in four environments, i.e., Beijing and Xunxian, China, in 2003 and 2004 (denoted as 2003BJ, 2004BJ, 2003XX, and 2004XX). Analysis of variance (ANOVA) was conducted in each environment by the VHP functionality in software package GAHP (Zhang et al., 2022b). Best linear unbiased predictions (BLUPs) were obtained across environments using Eq. 7 by R package lme4 for PIL and IF_2_, respectively.


, (7) $$y_{ijk}=\mu +G_{i}+E_{j}+R_{k\left(j\right)}+GE_{ij}+e_{ijk}$$

where *y_ijk_* represented the phenotypic value; *μ* was the overall mean; *G_i_* was the effect of genotype *i*; *E_j_* was the effect of the location-year combination (i.e., environment) *j*; *R_k_*_(_ *_j_*_)_ was the effect of replication *k* nested in environment *j*; *GE_ij_* was the G×E interaction between genotype *i* and environment *j*; and *e_ijk_* was the residual effect associated with genotype *i* in environment *j* and replication *k*.

BLUPs of EL were used in QTL detection by two mapping approaches, i.e., IF2 and IFL (**Table 3**). Scanning step and the probability for entering variables in stepwise regression were set to 1 cM and 0.001, respectively. Threshold LOD score was set at 3.00 for IF2, and 5.00 for IFL. QTLs identified by different mapping approaches were regarded as co-located, if their genetic distance was smaller than 5 cM. The detected QTLs were compared with the reported QTLs in database MaizeGDB (https://www.maizegdb.org/), according to the physical positions of flanking markers. If a detected QTL was located at the physical interval determined by flanking markers in the database, they were treated to be the same QTL.

### QTL distribution models in detection power simulation

Two simulation experiments were conducted to illustrate the efficiency of combined approach in mapping QTLs related to heterosis. Ten chromosomes were considered in simulation I, each of which was 100 cM in length. Twenty-one markers were evenly distributed on each chromosome, and the average distance between any two adjacent markers was 5 cM. One QTL was located at 22.5 cM on each of the first nine chromosomes, and their genetic effects and variances were given in **Table 4**. One thousand populations each of IB_1_, IB_2_ and IF_2_, derived from the PIL of DHs, were generated by genetic breeding simulation platform Blib, each with a size of 200 (Zhang et al., 2022a). The random error variance was set to 1. Additional one thousand populations each of IB_1_, IB_2_ and IF_2_ with a size of 200 were simulated under the null QTL model to estimate the empirical distribution of test statistic, and obtain the threshold LOD score. 1000 highest LOD scores from the 1000 simulated runs were sorted, and then the threshold LOD score was estimated by the 95% quantile, so as to control the genome-wide type I error below 0.05.

**Table 4:** Genetic effects and variances of the pre-defined QTLs in simulation experiment I.

QTL no.	Genetic effects		Degree of dominance (*d*/*a*)	Genetic variance			
	Additive (*a*)	Dominant (*d*)		PIL	IB_1_	IB_2_	IF_2_
1	0	0.5	NA	0	0.0625	0.0625	0.0625
2	0	1	NA	0	0.2500	0.2500	0.2500
3	0.5	-1	-2	0.2500	0.5625	0.0625	0.3750
4	0.5	-0.5	-1	0.2500	0.2500	0	0.1875
5	0.5	-0.25	-0.5	0.2500	0.1406	0.0156	0.1406
6	0.5	0	0	0.2500	0.0625	0.0625	0.1250
7	0.5	0.25	0.5	0.2500	0.0156	0.1406	0.1406
8	0.5	0.5	1	0.2500	0	0.2500	0.1875
9	0.5	1	2	0.2500	0.0625	0.5625	0.3750

Chromosome and marker information in simulation II was the same as the actual maize populations PIL and IF_2_. QTLs affecting EL detected by mapping approach IF2 were used as the pre-defined QTLs, and their genetic effects and variances were given in **Supplementary Table 4**. One thousand populations each of IB_1_ and IB_2_ with a size of 166 (same as the actual PIL), and one thousand populations of IF_2_ with a size of 157 (same as the actual IF_2_) were generated from the PIL of RILs. Random error variance was set to 1. Additional one thousand populations each of IB_1_ and IB_2_ with a size of 166, and one thousand populations of IF_2_ with a size of 157 were simulated under the null QTL model to obtain the threshold LOD score.

In both simulation experiments, scanning step, the probability for entering variables in stepwise regression, and length of the support interval were set to 1 cM, 0.001 and 10 cM, respectively. If a peak higher than threshold was observed within the support interval around the position of one pre-defined QTL, the peak is treated as a true positive. If the detected peaks are out of any support interval, they are considered to be false positives. When more than one peak occurred within the same interval, only the one with the highest LOD score is counted. Power of each pre-defined QTL is the ratio of true positives to 1000 simulation runs (Li et al., 2010). False discovery rate (FDR) is defined as the proportion of false positives to the total number of true and false positives (Benjamini and Hochberg, 1995).

## Results

### Results of the combined ANOVA from the maize PIL and IF_2_ populations

For EL in each environment, additive and dominant variances as well as the narrow-sense and broad-sense heritabilities calculated from ANOVA were shown in **Supplementary Table 5**. Additive variance varied from 1.67 to 1.91, which was the smallest in 2004XX and the largest in 2003XX. Dominant variance varied from 0.68 to 1.61, which was the smallest in 2004XX and the largest in 2003XX. Additive variance was higher than dominant variance in each of the four environments. Heritability in the narrow sense ranged from 0.43 to 0.51, which was the smallest in 2003XX and largest in 2004XX. Heritability in the broad sense ranged from 0.69 to 0.78, which was the smallest in 2003BJ and largest in 2003XX.

### QTLs identified from the maize PIL and IF_2_ populations

The LOD score profiles from the independent mapping IF2 and combined mapping IFL were displayed in **Supplementary Figures 1A, B**, respectively. Under the threshold LOD score of 3.00, seven QTLs were detected in population IF_2_, explaining 51.30% of the phenotypic variance in total, two on chromosome 5, and one each on chromosomes 1, 2, 4, 7 and 8. qEL8 had the largest LOD score at 9.35 and the largest percentage of variance explained (PVE) at 16.11%. Three QTLs detected in IF_2_ have been reported in previous studies, i.e., qEL1.1, qEL2 and qEL5.2, by alignment with the MaizeGDB database (**Table 5**).

**Table 5:** Mapping results for ear length in the actual maize PIL and IF_2_ populations.

Mapping approach	QTL name	Pos. (cM)	LeftCI (cM)	RightCI (cM)	LOD	LOD (A)^a^	LOD (D)^b^	PVE (%)	PVE.A (%)^c^	PVE.D (%)^d^	Add	Dom	Degree of dominance^e^	Co-localization^f^
IF2	qEL1.1	24	21.50	26.50	3.28			4.63			-0.43	0.47	D	qearl1
	qEL2	113	112.50	114.50	3.31			4.58			0.45	0.32	PD	qearl40
	qEL4	141	139.50	141.50	4.58			6.93			0.27	0.74	OD	
	qEL5.2	145	144.50	146.50	3.82			5.95			0.56	0.10	A	qearl9
	qEL5.3	230	227.50	230.50	4.85			7.60			0.56	-0.01	A	
	qEL7.1	41	34.50	43.50	3.12			5.50			0.47	0.19	PD	
	qEL8	199	196.50	201.50	9.35			16.11			-0.83	-0.19	PD	
IFL	qEL1.2	49	48.50	49.50	10.03	7.48	2.55	3.44	1.73	1.71	-0.40	0.43	D	qearl37
	qEL1.3	88	84.50	89.50	7.57	4.62	2.95	3.12	1.04	2.08	0.31	0.21	PD	qearl17
	qEL5.1	83	82.50	83.50	8.13	4.36	3.77	3.55	0.95	2.59	0.30	0.43	OD	qearl29
	qEL5.2	149	148.50	151.50	9.78	7.49	2.28	3.47	1.77	1.70	0.44	0.16	PD	qearl9
	qEL5.3	227	226.50	227.50	26.43	18.97	7.46	11.04	5.35	5.70	0.67	-0.12	A	
	qEL5.4	245	242.50	247.50	11.34	8.19	3.15	4.03	1.89	2.14	-0.42	-0.24	PD	
	qEL6.1	19	18.50	21.50	11.19	10.02	1.17	3.19	2.38	0.81	-0.48	0.03	A	qearl25
	qEL6.2	30	28.50	30.50	5.52	4.03	1.49	1.91	0.89	1.03	-0.04	0.41	OD	
	qEL7.2	66	63.50	68.50	11.58	8.41	3.17	4.20	2.03	2.18	0.43	0.36	D	
	qEL8	198	195.50	201.50	8.44	0.39	8.05	6.14	0.08	6.06	-0.03	-0.03	D	

LOD, logarithm of odds; PVE, percentage of phenotypic variance explained by individual QTL.

^a^ LOD score for additive effect. ^b^ LOD score for dominant effect. ^c^ Phenotypic variation explained by additive effect of the detected QTL.

^d^ Phenotypic variation explained by dominant effect of the detected QTL. ^e^ A, additive; PD, partial dominant; D, dominant; OD, over-dominant.

^f^ Co-localize with previously reported QTLs in database MaizeGDB (https://www.maizegdb.org/).

Under the threshold LOD score of 5.00, ten QTLs were identified by the combined mapping IFL, explaining 44.09% of the phenotypic variance in total, four on chromosome 5, two on chromosome 1, two on chromosome 6, and one each on chromosomes 7 and 8 (**Table 5**). qEL5.3 had the largest LOD score at 26.43 and the largest PVE at 11.04%. Five QTLs detected by IFL have been reported in previous studies, i.e., qEL1.2, qEL1.3, qEL5.1, qEL5.2 and qEL6.1, by alignment with the MaizeGDB database (**Table 5**). Three QTLs were detected by both independent and combined mapping approaches, i.e., qEL5.2, qEL5.3 and qEL8.

The degree of dominance is defined as the absolute value of the ratio of dominant to additive effects (i.e., |*d*/*a*|). QTLs can be classified into four categories according to the estimated degrees of dominance, i.e., additive (|*d*/*a*|<0.2), partial dominant (0.2≤|*d*/*a*|<0.8), dominant (0.8≤|*d*/*a*|<1.2), and over-dominant (|*d*/*a*|≥1.2) (Stuber et al., 1987). The mid-parental and higher-parental heterosis in percentages were ranged from –0.92% to 46.28% and -15.04% to 45.54%, respectively (**Supplementary Figure 2**). The average mid-parental and higher-parental heterosis were 24.40% and 17.15%, respectively. Among the 10 QTLs detected by combined mapping, 2 were additive, 3 partial dominant, 3 dominant, and 2 over-dominant. Three of the five dominant and over-dominant QTLs had positive dominant effects, leading to moderate heterosis on EL in the IF_2_ population.

### Power analysis and mapping results for simulation experiment I

**Figure 2:**
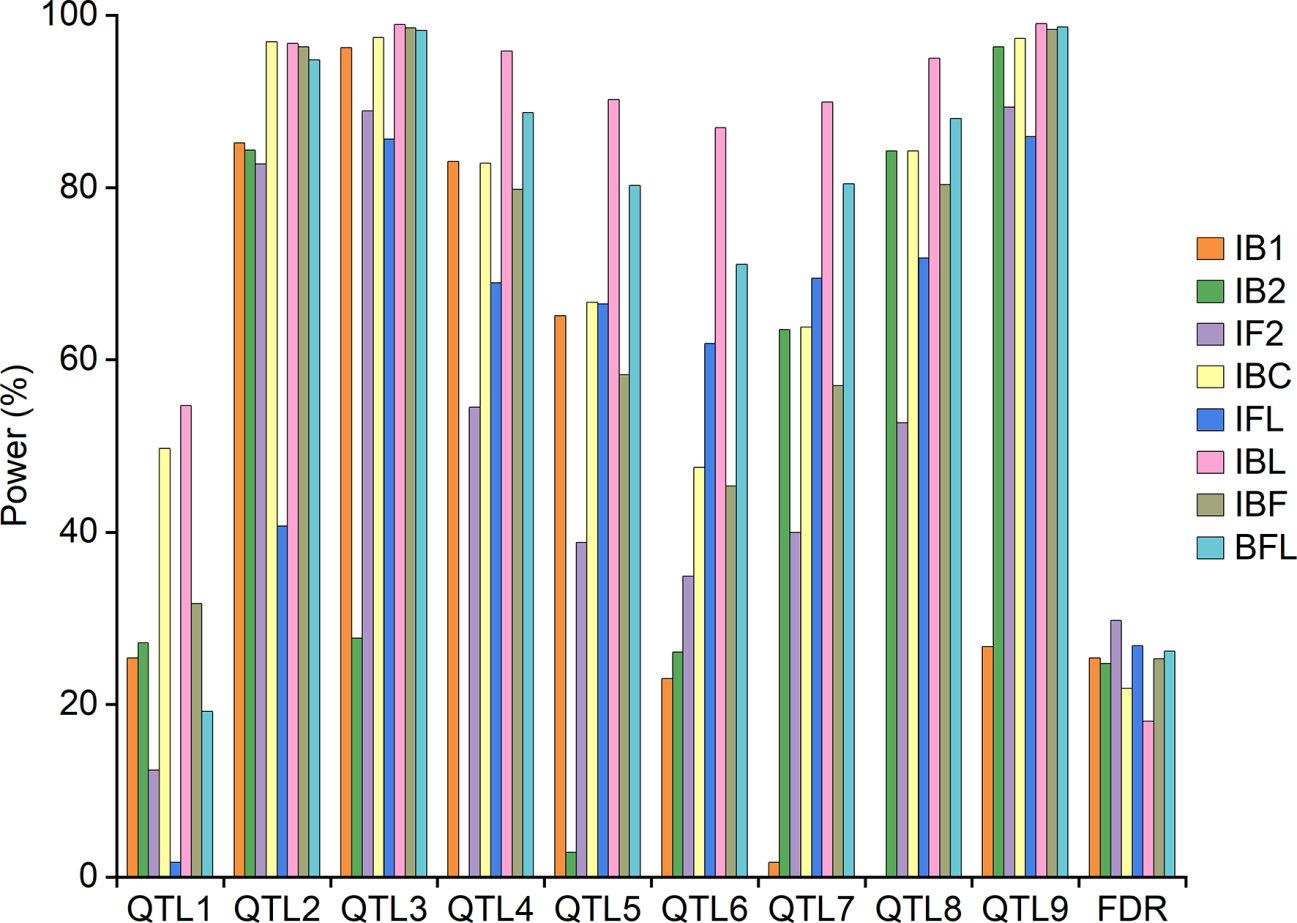
QTL detection power from individual and combined mappings in simulation experiment I.

Under the null-QTL model, the threshold LOD scores for different mapping approaches were determined and given in **Supplementary Table 6**. Detection power of each pre-defined QTL was shown in **Figure 2**, and the average power across all QTLs was shown in **Supplementary Figure 3A**. Detection power depends on the value of *a*-*d* in population IB_1_, and on the value of *a*+*d* in population IB_2_ (**Table 1**). Additive effects of QTL1 and QTL2 are equal to 0, and thus *a*-*d* and *a*+*d* are equal by absolute values; dominant effect of QTL6 is equal to 0, and thus *a*-*d* and *a*+*d* have same value. In other words, genetic variance of QTL1 was the same in populations IB_1_ and IB_2_. So were QTL2 and QTL6. Therefore, independent mappings IB1 and IB2 achieved similar detection power for QTL1, QTL2 and QTL6. For QTL3, QTL4 and QTL5, IB1 achieved much higher detection power than did IB2, as the additive and dominant effects were at different directions, making *a*-*d* much larger than *a*+*d*, and genetic variance in population IB_1_ larger than that in IB_2_. On the contrary, detection power from IB2 was much higher than that from IB1 for QTL7, QTL8 and QTL9, as the additive and dominant effects were at the same direction, making *a*+*d* much larger than *a*-*d*, and genetic variance in population IB_2_ larger than that in IB_1_ (**Table 4**; **Figure 2**).

Combined mapping IBL had similar or higher powers and lower FDR than did IBC, followed by independent mappings IB1 and IB2 (**Figure 2**). The average detection power from IBL was also higher than that from IBC, followed by IB1 and IB2 (**Supplementary Figure 3A**). Combined mapping IFL had higher powers than did IF2 for five QTLs, i.e., QTL4, QTL5, QTL6, QTL7 and QTL8. FDR from IFL was 2.9% lower than that from IF2 (**Figure 2**). The average detection power from IFL was 61.4%, which was 6.5% higher than that from IF2 (**Supplementary Figure 3A**). Combined mapping IBC achieved higher detection power and lower FDR than did IFL except for QTL6 and QTL7 (**Figure 2**). The average power from IBC was 76.3%, which was 14.9% higher than that from IFL (**Supplementary Figure 3A**).

Combined mapping BFL had higher powers than did IBF for six QTLs, i.e., QTL4, QTL5, QTL6, QTL7, QTL8 and QTL9. FDR from BFL was 0.86% higher than that from IBF (**Figure 2**). The average detection power from BFL was 8.2% higher than that from IBF (**Supplementary Figure 3A**). Both IBF and BFL performed similarly or better than did IBC, IFL and IBL for QTL3 and QTL9 (**Figure 2**). Average power from IBF was 10.3% higher than that from IFL. Average power from BFL was 3.7% and 18.5% higher than that from IBC and IFL (**Supplementary Figure 3A**).

Deviation between the estimated and predefined true positions, additive and dominant effects for the nine QTLs was given in **Supplementary Table 7**, averaged from the 1000 simulation runs. IB2 and IBL each achieved the highest accuracy on estimated positions for two QTLs; and IB1, IF2, IBC, IBF and BFL each achieved the highest accuracy for one QTL. The average deviation between the estimated and predefined positions from IB2 was the smallest, followed by IBL and IBC. Difference between the three approaches was minor. Additive and dominant effects cannot be separated by IB1 and IB2. IBC and IBL achieved the lowest deviations on estimated additive effects for four and three QTLs, respectively; IF2 and IFL each achieved the lowest bias on estimated additive effects for one QTL. IF2 and IBC performed the best on estimated dominant effects for four and three QTLs, respectively; IBF and BFL each achieved the lowest deviations on estimated dominant effect for one QTL. Average deviations from IBC on additive and dominant effects were the smallest among all mapping approaches (**Supplementary Table 7**).

### Power analysis and mapping results for simulation experiment II

**Figure 3:**
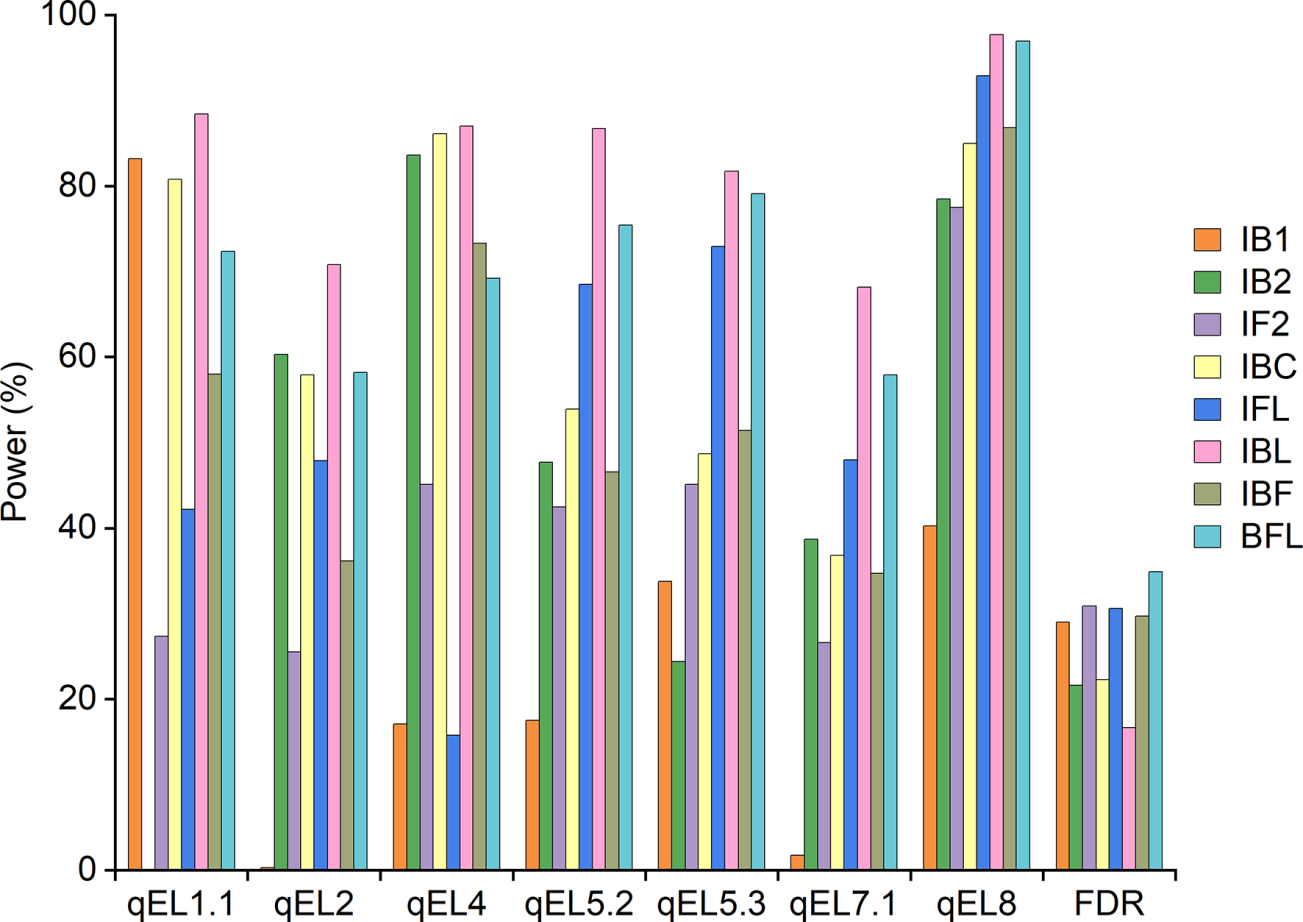
QTL detection power from individual and combined mappings in simulation experiment II.

The threshold LOD scores applied in simulation II were given in **Supplementary Table 6** for different mapping approaches. Seven QTLs detected in the maize population IF_2_ (**Table 5**) were used as the pre-defined QTLs. Detection powers were shown in **Figure 3**, and the average power across all QTLs from each mapping approach was provided in **Supplementary Figure 3B**. Independent mapping IB1 achieved much higher detection power than IB2 for qEL1.1, as qEL1.1 was a dominant QTL and its additive and dominant effects were at different directions. On the contrary, detection power from IB2 was much higher than that from IB1 for qEL2, qEL4, qEL7.1 and qEL8, as these QTLs were partial dominant or over-dominant, and their additive and dominant effects were at the same direction. Difference of powers between IB1 and IB2 was smaller for qEL5.2 and qEL5.3 than that for the other QTLs, both of which were additive QTLs, resulting in similar values between *a*+*d* and *a*-*d* (**Table 5**; **Figure 3**).

Combined mapping IBL achieved higher power and lower FDR than IBC. IBC achieved higher power than did IB1 and IB2 for four QTLs, and the FDR from IBC was similar or lower than that from IB1 and IB2 (**Figure 3**). The average power from combined mapping IBL was also higher than that from IBC, followed by IB1 and IB2 (**Supplementary Figure 3B**). Detection power from combined mapping IFL was higher than that from IF2, except for qEL4, and FDR from IFL was 0.27% lower than that from IF2 (**Figure 3**). Average power from IFL was 14.1% higher than that from IF2 (**Supplementary Figure 3B**). IBC achieved higher power than did IFL for qEL1.1, qEL2 and qEL4. FDR from IBC was 8.3% lower than that from IFL (**Figure 3**). Average power from IBC was 8.7% higher than that from IFL (**Supplementary Figure 3B**).

Combined mapping BFL had higher power for six QTLs than did IBF, but FDR from BFL was 5.12% higher than that from IBF (**Figure 3**). Average power from BFL was 17.4% higher than that from IBF (**Supplementary Figure 3B**). For each QTL, IBF and BFL had lower detection power than did IBC, IFL or IBL (**Figure 3**). But the average power of BFL was 8.6% and 17.3% higher than that from IBC and IFL, respectively (**Supplementary Figure 3B**). When three genotypes are included in mapping populations, detection powers of different QTLs can be hardly compared by their additive and dominant effects. In this case, genetic variance caused by each QTL is more useful. It has been properly used to quantify the effect of various segregation distortions on QTL mapping in F_2_ populations (Zhang et al., 2010). In **Figure 3**, different detection powers observed from different QTLs and mapping populations can be explained by genetic variance as well. Taking qEL1.1 as an example, its genetic variance was the smallest in population IB_2_, followed by IF_2_ and IB_1_. Its detection power was also the lowest by mapping IB2, followed by IF2 and IB1 (**Supplementary Table 4**).

**Supplementary Table 8** showed the deviation between the estimated and pre-defined QTL positions, additive and dominant effects in simulation II, averaged from the 1000 simulation runs. Combined mapping IFL had the highest accuracy on estimated positions for four QTLs; IB1 and IBL each achieved the highest accuracy for one and two QTLs. Average deviation between the estimated and predefined positions was the smallest from IBL, followed by IBC. IBC and IFL each performed the best on estimated additive effects for two QTLs; IF2, IBF and BFL each achieved the highest accuracy on estimated additive effect for one QTL. Average deviation of the estimated additive effect from IBC was 0.0592, which was the smallest among all mapping approaches. IBL and IBF each achieved the lowest bias on estimated dominant effects for two and three QTLs, respectively; IF2 and IBC each achieved the smallest deviation on estimated dominant effect for one QTL. Average deviation on estimated dominant effect from IF2 was the smallest, followed by IBL and IBF (**Supplementary Table 8**).

## Discussion

### Transformations after the phenotypic values are adjusted

In combined approaches as shown in this study, transformations were conducted after the phenotypic values were adjusted. Adjustment made by Eq. 6 not only assures the background genetic variations out of the current scanning interval are controlled, but also leaves solely the one-locus variation in the adjusted phenotypes. As shown in **Tables 1** and **2**, transformations given in Eqs. 1 to 4 are able to separate additive and dominant effects efficiently under the one-locus model. However, it should be noted that the theoretical results given in **Tables 1** and **2** cannot be simply extended to two or more QTLs. During our research, we have conducted the transformations first, and then used the transformed data as phenotypic values in QTL mapping. Reduced detection powers were observed, and the estimation of additive and dominant effects were more biased. In fact, when two QTLs are considered, additive, dominant and epistatic effects are confounded in the transformed values in populations IBC and IF_2_. On the other aspect, this may indicate that the transformations used to separate additive and dominant effects may no longer be suitable for mapping epistatic QTLs. The combined approach and algorithm for epistasis mapping through the two-dimensional genomic scanning needs further investigations.

### Properties and advantages of the combined mapping approach

Both simulation experiments indicated that the combined approaches IBL and IBC had higher detection powers and lower FDR than did individual population mapping IB1 and IB2. However, mapping efficiency depends on the populations used in combined mapping. IBL had higher detection power than did IBC for all pre-defined QTLs (**Figures 2**, **3**; **Supplementary Figure 3**). Compared with IF2, IFL had higher detection power for additive, partial dominant and dominant QTLs. Detection power from IBC was significantly higher than that from IFL for QTLs with dominant or over-dominant effects and QTLs without additive effects, which are more important in heterosis studies (**Figures 2**, **3**). BFL performed better than did IBF for additive, partial dominant and dominant QTLs (**Figures 2**, **3**). IBL and IBC performed better on estimated additive and dominant effects than did the other methods (**Supplementary Tables 7**, **8**).

Combined mapping showed greater advantages in IBC populations than did in IF_2_, due to the present of fewer genotypes. More genotypes and genetic effects associated with IF_2_ may complicate the building of genotype to phenotype model, and then affect the efficiency of background control in QTL mapping. In addition, the IBC populations are generated by backcrossing of PIL with the two original parents. One line in PIL corresponds to exact one individual in either IB_1_ or IB_2_. However, sampling of pure lines in PIL is needed to generate IF_2_, which may cause the random drift in gene frequencies in IF_2_. For this reason, IBC population may be considered firstly when using the immortalized heterozygous populations in genetic study. In addition, to reduce the random effects in the combined analysis, different populations should be grown under the same set of environmental conditions.

### Simultaneous use of heterozygous and homozygous populations to enhance our understanding of heterosis

Investigating the genetic mechanism of heterosis is of great importance in hybrid breeding and agriculture production. The detection of heterotic loci and estimation of heterotic effects require genetic populations containing both heterozygous and homozygous genotypes. IBC and IF_2_ are considered as ideal populations for the comprehensive dissection of heterosis. Up to now, there are few complete collections of IBC and IF_2_ populations which are derived from the same two homozygous parents. Li et al. (2018a); Li et al. (2018b) present such an example in cotton using two elite upland cotton germplasms HS46 and MARCABUCAG8US-1-88. Simulations in this study indicated that the detection power from IBF was higher than that from IF2, and the detection power from BFL was higher than that from IFL (**Supplementary Figure 3**). In other words, compared with using IF_2_ solely, the combined mapping using populations IBC and IF_2_ can improve the QTL detection power. Li et al. (2018a) also indicated that the combination of IBC and IF_2_ can cover more heterozygous loci and identify more QTLs than individual populations.

The combined QTL mapping approach proposed in this study has been implemented in integrated software package called GAHP (Zhang et al., 2022b). There are four functionalities in GAHP V1.0, i.e., (1) MHP: drawing of genetic linkage map; (2) VHP: ANOVA and estimation of heritability on phenotypic observations; (3) QHP: QTL mapping with bi-parental immortalized heterozygous populations; (4) SHP: simulation of bi-parental immortalized populations and power analysis of QTL detection. With the integrated software package GAHP (Zhang et al., 2022b), we trust that the mapping approach provided in this study will facilitate the efficient use of immortalized heterozygous populations in genetic studies. It will enhance the investigation on the molecular mechanism of heterosis, and finally contribute to the improved efficiency of hybrid breeding programs in plants.

## Data availability statement

The original contributions presented in the study are included in the article/**Supplementary Material**. Further inquiries can be directed to the corresponding authors.

## Author contributions

XH conducted the simulation study and data analysis. LZ and JW conceived and designed the research, and proposed and developed the combined mapping approach. All authors contributed to the article and approved the submitted version.
