# Supplementary material for: Combined QTL mapping on bi-parental immortalized heterozygous populations to detect the genetic architecture on heterosis
Source: Front Plant Sci. 2023 Apr 4;14:1157778. doi: 10.3389/fpls.2023.1157778 (PMC10112513; doi:10.3389/fpls.2023.1157778)
Supplement: Supplementary file 2 [file DataSheet_2.docx]

Supplementary Material

Combined QTL mapping on bi-parental immortalized heterozygous populations to detect the genetic architecture on heterosis

**Xuexue Huo^1^, Jiankang Wang^1,2*^, Luyan Zhang^1^**^*^

* Correspondence: wangjiankang@caas.cn, zhangluyan@caas.cn

# Supplementary Figures and Tables

## Supplementary Figures


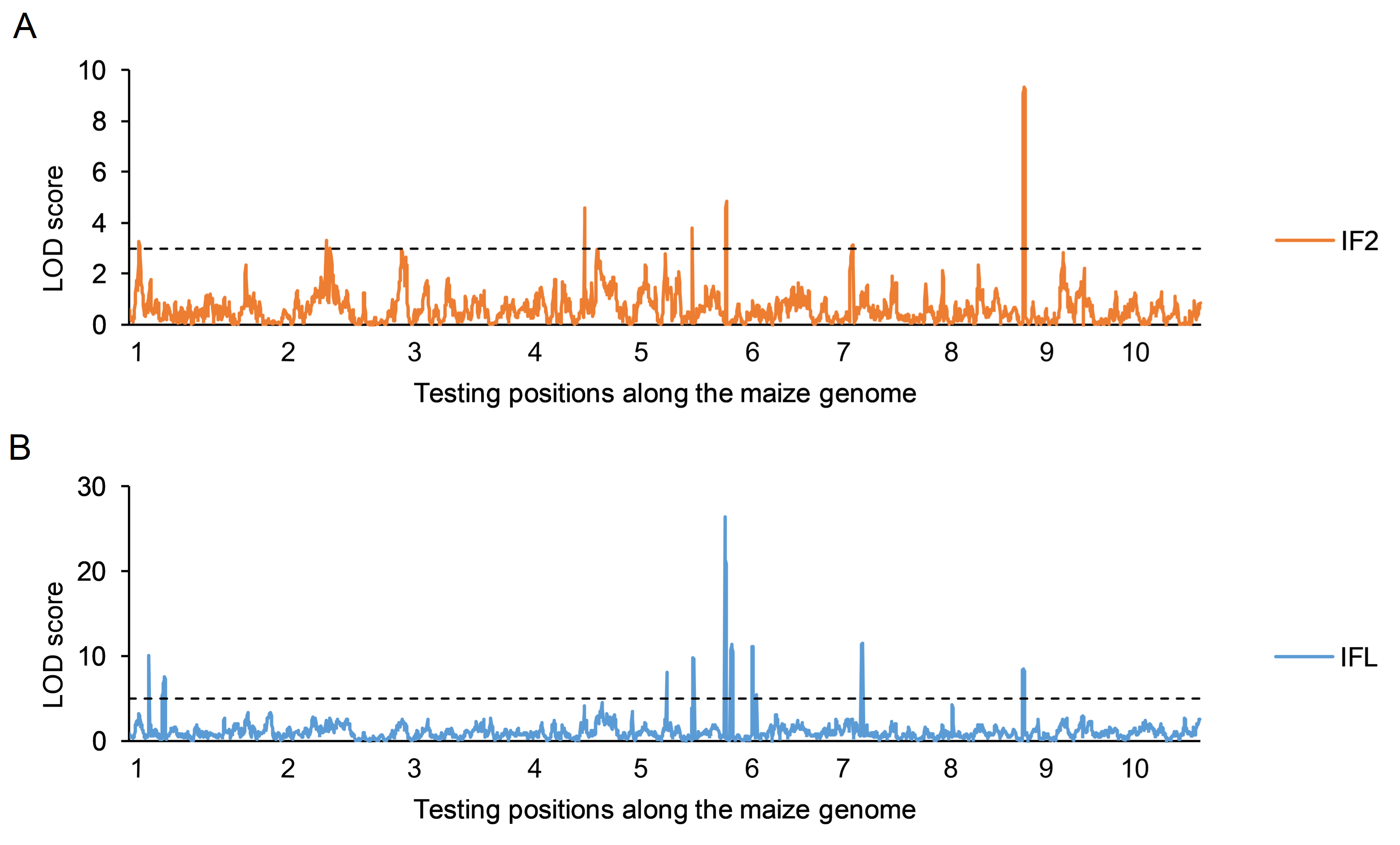


**Supplementary Figure 1.** LOD score of ear length in the actual PIL and IF_2_ populations in maize


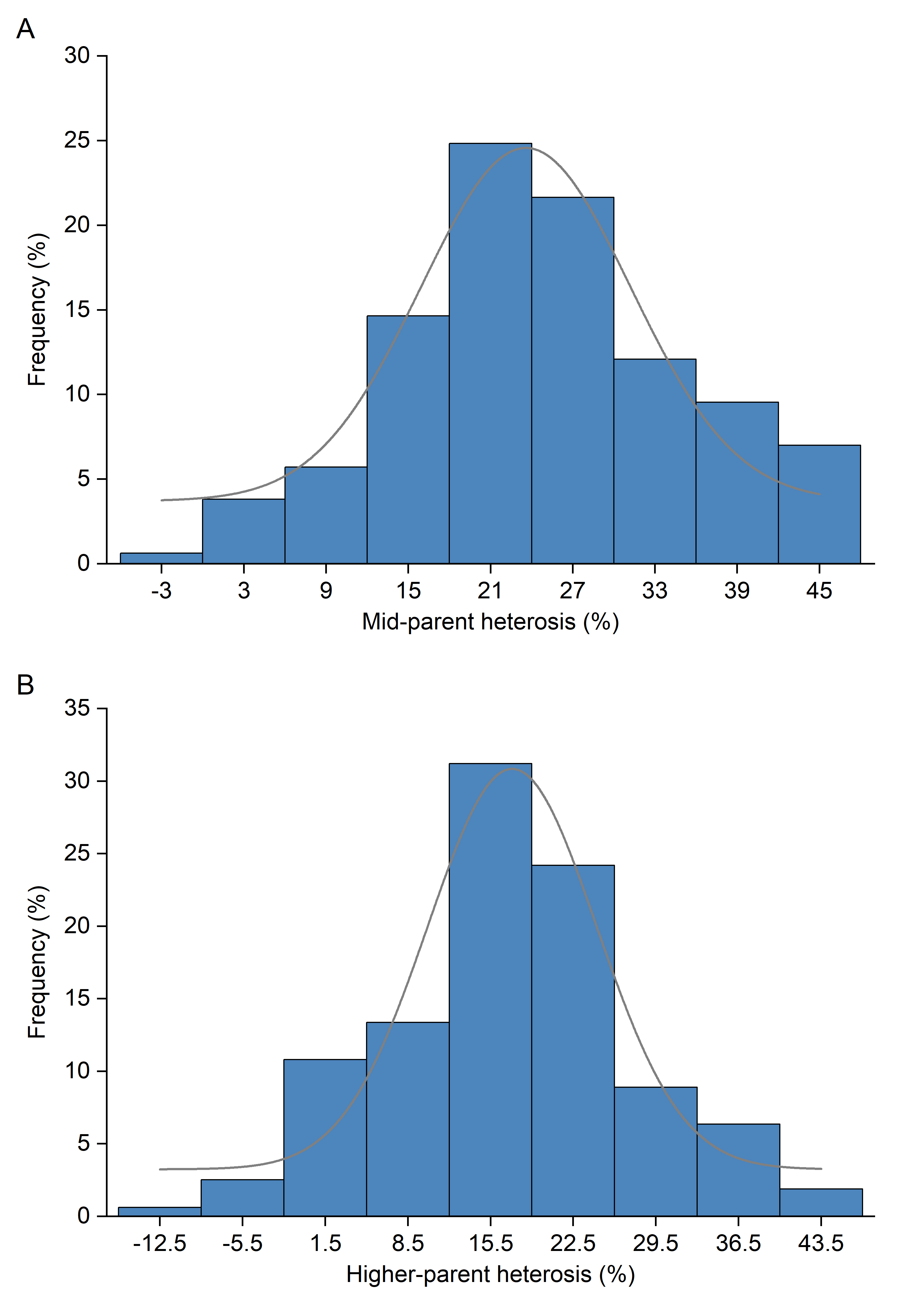


**Supplementary Figure 2.** The distribution of mid-parental and higher-parental heterosis for ear length in the actual PIL and IF_2_ populations in maize


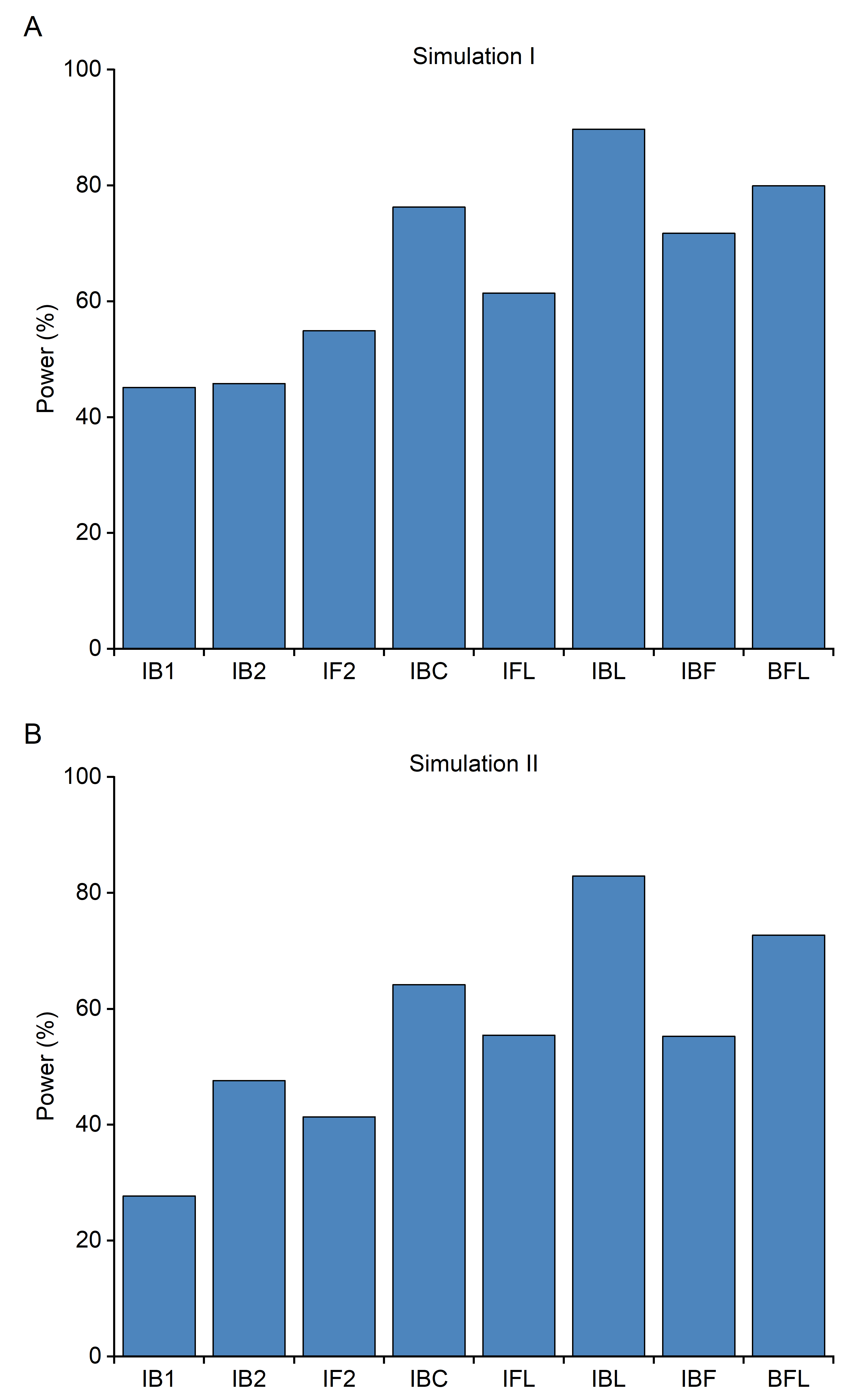


**Supplementary Figure 3.** The average power across all QTLs in two simulation experiments

## Supplementary Tables

**Supplementary Table 1** Genotypes and their frequencies at two linked loci in populations PIL, IB_1_ and IB_2_ from two types of bi-parental pure lines

| Population | | | Type of PIL | |
| --- | --- | --- | --- | --- |
| PIL | IB_1_ | IB_2_ | F_1_-derived DHs | RILs derived by repeated selfing |
| *AABB* | *AABB* | *AaBb* | $\frac{1}{2}(1-r)$ | $\frac{1}{2}(1-R)$ |
| *AAbb* | *AABb* | *Aabb* | $\frac{1}{2}r$ | $\frac{1}{2}R$ |
| *aaBB* | *AaBB* | *aaBb* | $\frac{1}{2}r$ | $\frac{1}{2}R$ |
| *aabb* | *AaBb* | *aabb* | $\frac{1}{2}(1-r)$ | $\frac{1}{2}(1-R)$ |

Notes: assume the genotypes of two original homozygous parents are *AABB* and *aabb* at two linked loci; *r* is the one-meiosis recombination frequency; *R* is the accumulated recombination frequency during repeated selfing; relationship between *r* and *R* is $R=\frac{2r}{1+2r}$.

**Supplementary Table 2** Genotypic frequencies at two linked loci in population IF_2_ from two types of bi-parental pure lines

| Genotype | Type of PIL | |
| --- | --- | --- |
|  | F_1_-derived DHs | RILs derived by repeated selfing |
| *AABB* | $\frac{1}{4}{(1-r)}^{2}$ | $\frac{1}{4}{(1-R)}^{2}$ |
| *AABb* | $\frac{1}{2}r(1-r)$ | $\frac{1}{2}R(1-R)$ |
| *AAbb* | $\frac{1}{4}r^{2}$ | $\frac{1}{4}R^{2}$ |
| *AaBB* | $\frac{1}{2}r(1-r)$ | $\frac{1}{2}R(1-R)$ |
| *AaBb* | $\frac{1}{2}(1-2r+2r^{2})$ | $\frac{1}{2}(1-2R+2R^{2})$ |
| *Aabb* | $\frac{1}{2}r(1-r)$ | $\frac{1}{2}R(1-R)$ |
| *aaBB* | $\frac{1}{4}r^{2}$ | $\frac{1}{4}R^{2}$ |
| *aaBb* | $\frac{1}{2}r(1-r)$ | $\frac{1}{2}R(1-R)$ |
| *aabb* | $\frac{1}{4}{(1-r)}^{2}$ | $\frac{1}{4}{(1-R)}^{2}$ |

Notes: assume the genotypes of two original homozygous parents are *AABB* and *aabb* at two linked loci; *r* is the one-meiosis recombination frequency; *R* is the accumulated recombination frequency during repeated selfing; relationship between *r* and *R* is$R=\frac{2r}{1+2r}$.

**Supplementary Table 3** Relationship between different LOD scores in combined mapping

| Method | LOD | LOD_PIL_^c^ | $\mathrm{LOD}_{\mathrm{IF}_{2}}$^d^ | LOD*_S_*^e^ | LOD*_T_*^f^ | LOD*_M_*^g^ | LOD*_H_*^h^ |
| --- | --- | --- | --- | --- | --- | --- | --- |
| IBC | LOD_A_^a^ | 0 | 0 | 1 | 0 | 0 | 0 |
|  | LOD_D_^b^ | 0 | 0 | 0 | 1 | 0 | 0 |
| IFL | LOD_A_ | 0 | 0 | 0 | 0 | 1 | 0 |
|  | LOD_D_ | 0 | 0 | 0 | 0 | 0 | 1 |
| IBL | LOD_A_ | $\frac{1}{2}$ | 0 | $\frac{1}{2}$ | 0 | 0 | 0 |
|  | LOD_D_ | 0 | 0 | 0 | 1 | 0 | 0 |
| IBF | LOD_A_ | 0 | $\frac{1}{3}$ | $\frac{2}{3}$ | $-\frac{1}{3}$ | 0 | 0 |
|  | LOD_D_ | 0 | $\frac{1}{3}$ | $-\frac{1}{3}$ | $\frac{2}{3}$ | 0 | 0 |
| BFL | LOD_A_ | 0 | 0 | $\frac{1}{2}$ | 0 | $\frac{1}{2}$ | 0 |
|  | LOD_D_ | 0 | 0 | 0 | $\frac{1}{2}$ | 0 | $\frac{1}{2}$ |

^a^ LOD score for additive effect.

^b^ LOD score for dominant effect.

^c^ LOD score from population PIL.

^d^ LOD score from population IF_2_.

^e^ LOD score from the summation transformation on populations IB_1_ and IB_2_.

^f^ LOD score from the subtraction transformation on populations IB_1_ and IB_2_.

^g^ LOD score from the mid-parental values.

^h^ LOD score from the mid-parental heterosis.

**Supplementary Table 4** Genetic effects and variances of pre-defined QTLs in simulation experiment II

| QTL name | Genetic effects | | Degree of dominance (*d*/*a*) | Genetic variance | | | |
| --- | --- | --- | --- | --- | --- | --- | --- |
|  | Additive (*a*) | Dominant (*d*) |  | PIL | IB_1_ | IB_2_ | IF_2_ |
| qEL1.1 | -0.43 | 0.47 | -1.10 | 0.1852 | 0.2039 | 0.0005 | 0.1485 |
| qEL2 | 0.45 | 0.32 | 0.71 | 0.2029 | 0.0042 | 0.1487 | 0.1272 |
| qEL4 | 0.27 | 0.74 | 2.73 | 0.0731 | 0.0547 | 0.2542 | 0.1727 |
| qEL5.2 | 0.56 | 0.10 | 0.19 | 0.3096 | 0.0512 | 0.1090 | 0.1575 |
| qEL5.3 | 0.56 | -0.01 | -0.02 | 0.3104 | 0.0803 | 0.0749 | 0.1552 |
| qEL7.1 | 0.47 | 0.19 | 0.41 | 0.2204 | 0.0195 | 0.1088 | 0.1193 |
| qEL8 | -0.83 | -0.19 | 0.23 | 0.6831 | 0.1025 | 0.2564 | 0.3502 |

**Supplementary Table 5** Estimated variance components and heritability of ear length using populations PIL and IF_2_ in maize

| Environment^a^ | Variance components | | | Heritability^b^ | |
| --- | --- | --- | --- | --- | --- |
|  | Additive | Dominant | Random error | Narrow-sense | Broad-sense |
| 2003BJ | 1.74 | 0.80 | 1.15 | 0.47 | 0.69 |
| 2003XX | 1.91 | 1.61 | 0.98 | 0.43 | 0.78 |
| 2004BJ | 1.69 | 1.06 | 0.95 | 0.46 | 0.74 |
| 2004XX | 1.67 | 0.68 | 0.95 | 0.51 | 0.71 |

^a^ 2003BJ and 2004BJ represented 2003 and 2004 Beijing; 2003XX and 2004XX represented 2003 and 2004 Xunxian..

^b^ Heritability was calculated by software package GAHP.

**Supplementary Table 6** Threshold LOD scores of individual and combined mappings in two simulation experiments

| Name of mapping | Simulation I | Simulation II |
| --- | --- | --- |
| IB1 | 2.74 | 3.38 |
| IB2 | 2.68 | 3.51 |
| IF2 | 3.59 | 4.34 |
| IBC | 3.46 | 4.21 |
| IBL | 3.04 | 3.74 |
| IFL | 7.02 | 8.32 |
| IBF | 3.51 | 4.08 |
| BFL | 4.44 | 5.07 |

**Supplementary Table 7** The deviation between the estimated values and pre-defined position, additive and dominant effects in simulation experiment I

| Parameter | QTL | IB1 | IB2 | IF2 | IBC | IFL | IBL | IBF | BFL |
| --- | --- | --- | --- | --- | --- | --- | --- | --- | --- |
| Position (cM)^a^ | QTL1 | 0.0669 | 0.0368 | 0.2016 | 0.0493 | 1.2059 | 0.0832 | 0.0268 | **0.0208** |
|  | QTL2 | 0.0458 | **0.0101** | 0.1143 | 0.0222 | 0.0848 | 0.0564 | 0.0119 | 0.1013 |
|  | QTL3 | 0.0873 | 0.0271 | 0.0411 | 0.0903 | 0.1425 | **0.0096** | 0.0391 | 0.0509 |
|  | QTL4 | 0.0855 | NA | 0.0339 | 0.0882 | 0.0776 | 0.0251 | **0.0013** | 0.0795 |
|  | QTL5 | 0.0223 | 0.0517 | 0.0206 | **0.0187** | 0.0729 | 0.0987 | 0.0317 | 0.0399 |
|  | QTL6 | **0.0087** | 0.159 | 0.1246 | 0.0516 | 0.1704 | 0.0167 | 0.1498 | 0.1315 |
|  | QTL7 | 0.2059 | 0.0764 | 0.0125 | 0.0972 | 0.0755 | **0.0017** | 0.2298 | 0.2425 |
|  | QTL8 | 4.5 | 0.1152 | **0.1072** | 0.152 | 0.2493 | 0.1705 | 0.1289 | 0.2182 |
|  | QTL9 | 0.0318 | **0.0016** | 0.0767 | 0.0262 | 0.2416 | 0.0768 | 0.1195 | 0.1633 |
|  | Mean | 0.5616 | **0.0597** | 0.0814 | 0.0662 | 0.2578 | 0.0599 | 0.0821 | 0.1164 |
| Additive effect^a^ | QTL1 |  |  | 0.0248 | 0.0052 | 0.009 | **0.0004** | 0.0094 | 0.008 |
|  | QTL2 |  |  | 0.0006 | 0.0021 | **0** | 0.0016 | 0.001 | 0.0007 |
|  | QTL3 |  |  | 0.123 | 0.0462 | 0.0605 | **0.021** | 0.0673 | 0.0379 |
|  | QTL4 |  |  | 0.0022 | **0.0006** | 0.0088 | 0.0583 | 0.0747 | 0.0747 |
|  | QTL5 |  |  | 0.0749 | **0.0028** | 0.0195 | 0.0645 | 0.05 | 0.0745 |
|  | QTL6 |  |  | 0.1024 | **0.0102** | 0.014 | 0.0762 | 0.0559 | 0.0916 |
|  | QTL7 |  |  | 0.0737 | **0.0114** | 0.0139 | 0.0665 | 0.0578 | 0.0762 |
|  | QTL8 |  |  | **0.0035** | 0.0043 | 0.0041 | 0.0626 | 0.079 | 0.0758 |
|  | QTL9 |  |  | 0.1259 | 0.0361 | 0.0508 | **0.0251** | 0.0657 | 0.0346 |
|  | Mean |  |  | 0.0590 | **0.0132** | 0.0201 | 0.0418 | 0.0512 | 0.0527 |
| Dominant effect^a^ | QTL1 |  |  | 0.2443 | 0.0082 | 0.3503 | 0.0175 | 0.0216 | **0.0031** |
|  | QTL2 |  |  | **0.0133** | 0.1115 | 0.1147 | 0.1139 | 0.1667 | 0.1618 |
|  | QTL3 |  |  | **0.0352** | 0.0846 | 0.1249 | 0.0949 | 0.1526 | 0.1667 |
|  | QTL4 |  |  | 0.0225 | **0.0042** | 0.1056 | 0.0423 | 0.0704 | 0.1007 |
|  | QTL5 |  |  | 0.0206 | **0.0174** | 0.0661 | 0.0422 | 0.0265 | 0.0518 |
|  | QTL6 |  |  | 0.0068 | 0.0063 | 0.0121 | 0.0067 | **0.0027** | 0.0068 |
|  | QTL7 |  |  | **0.0317** | 0.0344 | 0.0711 | 0.0376 | 0.038 | 0.0654 |
|  | QTL8 |  |  | 0.0398 | **0.0029** | 0.1229 | 0.0387 | 0.0691 | 0.0977 |
|  | QTL9 |  |  | **0.0251** | 0.0915 | 0.1016 | 0.1051 | 0.1482 | 0.1666 |
|  | Mean |  |  | 0.0488 | **0.0401** | 0.1188 | 0.0554 | 0.0773 | 0.0912 |

^a^ Estimated position and genetic effects were averaged from 1000 simulated runs. Accuracy of a method was measured by the deviation between the estimated and pre-defined values of positions and genetic effects. The method with the smallest deviation was regarded as the best and bolded.

**Supplementary Table 8** The deviation between the estimated values and pre-defined position, additive and dominant effects in simulation experiment II

| Parameter | QTL | IB1 | IB2 | IF2 | IBC | IFL | IBL | IBF | BFL |
| --- | --- | --- | --- | --- | --- | --- | --- | --- | --- |
| Position (cM)^a^ | qEL1.1 | 0.1142 | 3 | 0.4708 | 0.1188 | 0.4739 | **0.0939** | 0.2328 | 0.2845 |
|  | qEL2 | 0.3333 | 0.1012 | 0.0667 | 0.1330 | **0.0063** | 0.1356 | 0.0884 | 0.1323 |
|  | qEL4 | 0.1754 | 0.0658 | 0.2727 | 0.1231 | **0.0443** | 0.0954 | 0.2019 | 0.1445 |
|  | qEL5.2 | 0.1143 | 0.0755 | 0.1153 | 0.0278 | **0.0190** | 0.0231 | 0.2361 | 0.0782 |
|  | qEL5.3 | 0.1775 | 0.1025 | 0.1463 | 0.0842 | 0.1372 | **0.0783** | 0.1654 | 0.1479 |
|  | qEL7.1 | 0.6111 | 0.1912 | 0.0188 | 0.1467 | **0.0167** | 0.0689 | 0.1182 | 0.0380 |
|  | qEL8 | **0.0968** | 0.2968 | 0.2877 | 0.2753 | 0.2852 | 0.1812 | 0.3134 | 0.2549 |
|  | Mean | 0.2318 | 0.5476 | 0.1969 | 0.1298 | 0.1404 | **0.0966** | 0.1937 | 0.1543 |
| Additive effect^a^ | qEL1.1 |  |  | 0.0507 | **0.0219** | 0.0809 | 0.0982 | 0.0464 | 0.0658 |
|  | qEL2 |  |  | 0.1361 | **0.0372** | 0.0839 | 0.1126 | 0.0472 | 0.0962 |
|  | qEL4 |  |  | 0.0855 | 0.0913 | 0.0802 | 0.0255 | 0.0333 | **0.0167** |
|  | qEL5.2 |  |  | 0.1142 | 0.0339 | **0.0315** | 0.1439 | 0.0954 | 0.1505 |
|  | qEL5.3 |  |  | 0.1244 | 0.0340 | **0.0321** | 0.1333 | 0.0759 | 0.1243 |
|  | qEL7.1 |  |  | 0.1649 | 0.0312 | 0.0611 | 0.0694 | **0.0244** | 0.0740 |
|  | qEL8 |  |  | **0.0099** | 0.1651 | 0.0636 | 0.2074 | 0.2113 | 0.2340 |
|  | Mean |  |  | 0.0980 | **0.0592** | 0.0619 | 0.1129 | 0.0763 | 0.1088 |
| Dominant effect^a^ | qEL1.1 |  |  | **0.0002** | 0.0142 | 0.1317 | 0.0316 | 0.0531 | 0.1016 |
|  | qEL2 |  |  | 0.0667 | 0.0537 | 0.1031 | 0.0619 | **0.0457** | 0.0947 |
|  | qEL4 |  |  | 0.1164 | **0.1036** | 0.1124 | 0.1082 | 0.2056 | 0.2378 |
|  | qEL5.2 |  |  | 0.0246 | 0.0745 | 0.0390 | 0.0647 | **0.0176** | 0.0419 |
|  | qEL5.3 |  |  | 0.0035 | 0.0414 | 0.0041 | **0.0019** | 0.0148 | 0.0099 |
|  | qEL7.1 |  |  | 0.0236 | 0.0817 | 0.0576 | 0.0336 | **0.0189** | 0.0420 |
|  | qEL8 |  |  | 0.0564 | 0.0642 | 0.0684 | **0.0130** | 0.0394 | 0.0490 |
|  | Mean |  |  | **0.0416** | 0.0619 | 0.0738 | 0.0450 | 0.0564 | 0.0824 |

^a^ Estimated positions and genetic effects were averaged from 1000 simulated runs. Accuracy of a method was measured by the deviation between estimated and pre-defined values of positions and genetic effects. The method with the smallest deviation was regarded as the best and bolded.
